# Emerging strategies for the development of food industries

**DOI:** 10.1080/21655979.2019.1682109

**Published:** 2019-10-30

**Authors:** Cristóbal N. Aguilar, Hector A. Ruiz, Anilú Rubio Rios, Mónica Chávez-González, Leonardo Sepúlveda, Rosa M. Rodríguez-Jasso, Araceli Loredo-Treviño, Adriana C. Flores-Gallegos, Mayela Govea-Salas, Juan A. Ascacio-Valdes

**Affiliations:** a Bioprocesses and Bioproducts Research Group, Food Research Department, School of Chemistry, Autonomous University of Coahuila, Saltillo, Mexico; b Biorefinery Group, Food Research Department, School of Chemistry, Autonomous University of Coahuila, Saltillo, Mexico

**Keywords:** Food industry, food preservation, green technologies, bioengineered, bioinformatics, strategies

## Abstract

Undoubtedly, the food industry is undergoing a dynamic process of transformation in its continual development in order to meet the requirements and solve the great problems represented by a constantly growing global population and food claimant in both quantity and quality. In this sense, it is necessary to evaluate the technological trends and advances that will change the landscape of the food processing industry, highlighting the latest requirements for equipment functionality. In particular, it is crucial to evaluate the influence of sustainable green biotechnology-based technologies to consolidate the food industry of the future, today, and it must be done by analyzing the mega-consumption trends that shape the future of industry, which range from local sourcing to on-the-go food, to an increase in organic foods and clean labels (understanding ingredients on food labels). While these things may seem alien to food manufacturing, they have a considerable influence on the way products are manufactured. This paper reviews in detail the conditions of the food industry, and particularly analyzes the application of emerging technologies in food preservation, extraction of bioactive compounds, bioengineering tools and other bio-based strategies for the development of the food industry.

## Graphical Abstract

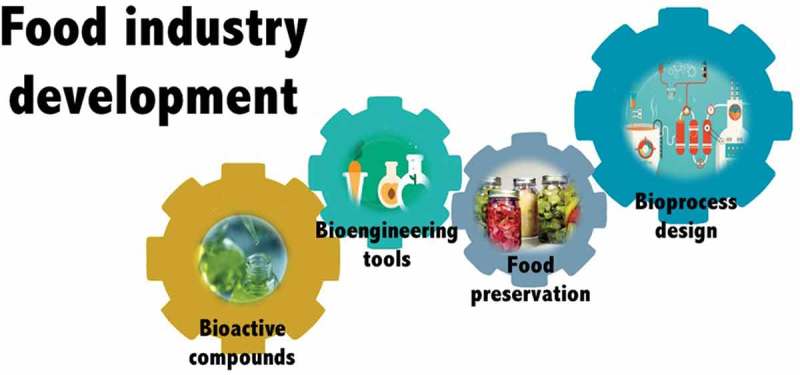

## 1. Introduction

Today, paying attention to consumer needs is key to the success of the food industry. This often involves reformulating products by using healthier sustainable ingredients, adding proteins, vitamins and antioxidants to foods, and labeling products as allergen-free, gluten-free, non-GMO, – in another case, organic and antibiotic-free. Also, caloric carbohydrates are increasingly eliminated or reduced in food, while measures are being taken to extend the shelf life of food and to prevent the filtration of counterfeit products in the supply chain. If there is a need for a recall, manufacturers need to act responsibly and proactively.

Considering the new conditions of the food market, the industrial sector has to focus on the need to incorporate more flexible equipment based on bioengineering, automation and robotics, in order to efficiently execute the line production. This approach will promote the channels and technology demanded by consumers, which will bring significant changes in the manufacture and supply of food demanded by the population.

Another critical consideration is the regulation of Food Safety Modernization. Beyond flexibility, food safety and safety is a priority, capable of conducting a highly selective classification to reduce food waste using smaller batch processing machinery to reduce and/or conserve energy consumption, even with inspection equipment for the detection and separation of ever-smaller foreign particles. The new technology that will underpin the food industry needs to be modernized in such a way that it is agile, easy to adapt to the minor changes in the market in real-time, focused on strict, sustainable cleaning and must be handled by staff and highly trained engineers, especially if it comes to biotechnology. From a technological perspective, that translates into faster reading times for vision equipment, self-diagnosis for preventive maintenance, and more connectivity and interoperability between systems, assuring food safety, traceability and authenticity.

Companies are also looking for the Industrial Internet of Things (IIoT) and data collection to better manage production schedules, resources, labor, and maintenance. With a growing and demanding population, it is a challenge to operate on the right scale many of the strategies and tools that will be capital-intensive solutions, including, in a strategy, the participation of human capital in the development of its capacity technological or financial capital to provide an efficient food experience to each of the communities it serves. This may include the production of genetically modified (GM) crops in inland agriculture, and throughout the course of over the next 10 years we see a position where sensors, algorithms, data fusion, machine perception and robotics come to eliminate many of the jobs and performance limitations of existing outdoor growing methods. It will be a reality the existence of smaller and automated warehouses, operating closer to the city centres, with goods fulfilled through small autonomous electric vehicles capable of delivering fresh food to smaller traders or become shops. Advances in bioscience, biotechnology and bioengineering and materials science in conjunction with changes in customer preferences will have an impact on how and where our food is produced.

The bioengineering represents an important impulse for the production of food bio-products and ingredients with and significant advantage in the high nutritional quality of new functional and intelligent foods, promoting the sustainability of the traditional and emerging food technologies. This review highlights the advantages and current improvement in the tools and strategies of bioengineering of microbes, animals and plants of relevance in the food industry, outlining the feasible approaches or opportunities for a modern food processor. The critical analysis results revealed that such bioengineered tools are essentials for the development of new enriched foods.

## 2. Trends in food processing

### 2.1. Application of emerging technologies in food preservation

Since food is harvested, it undergoes physical, chemical or biological changes that cause it to deteriorate. Food preservation is a methodology that avoids contamination with microorganisms. These microorganisms, in addition to enzymes, are the main agents responsible for change and should therefore be the targets of conservation techniques [1]. Some of the newer emerging technologies for food conservation, to reduce or eliminate the number of important pathogenic microorganisms in food, and/or for the extraction of bioactive compounds that are useful in the food industry, are listed below.

#### 2.1.1. High hydrostatic pressure

The high hydrostatic pressure (HHP) methodology is mainly used to physically and chemically modify any chemical compound present to benefit the quality of the food; it is also known as cold pasteurization or pressurization. This technology is of great interest in the food industry for its effectiveness in preserving food, which makes it superior to conventional thermal processes [2], because the latter inevitably cause a loss of nutritional quality and sensory attributes. Among the alternative (non-thermal) treatments currently known for food preservation (high intensity electrical pulses, oscillating magnetic fields, high intensity luminous pulses and ultrasound), HHP is considered the most viable technique from a commercial point of view [3] and the one that has demonstrated effectiveness in the inactivation of bacterial spores and enzymes [4]. Among the advantages offered by treatment with HHP over other non-thermal technologies, they can be mentioned:

- The HHP treatment avoids the alteration of the food, by the pressure transmitted uniformly and suddenly to the system, that is to say, there are no pressure gradients. Unlike thermal processes, HHP treatment does not depend on the volume and shape of the sample, decreasing the time required to process large quantities of food [5].
- HHP does not cause degradation of thermolabile nutrients such as vitamins (being a low temperature technology), and does not modify the activity or presence of low molecular weight compounds, such as those responsible for the aroma and taste of food, compared to traditional methods of pasteurization [6].

By treating foods by high hydrostatic pressure, they retain their nutritional properties and organoleptic characteristics; that is, there is no considerable loss of properties compared to a fresh product.6 The preservation of the quality and freshness of HHP-processed foods is due to the fact that the pressure-temperature-time conditions used produce small chemical changes, while the sensory and nutritional properties are not altered. Different studies have proven that products processed by high pressure maintain the characteristics of a fresh product, so that in most cases it is difficult to identify them from a fresh untreated product [3,4]. The HHP system allows the destruction of microorganisms that cause the degradation and loss of the quality of diverse foods, generally pressures between 100 to 800 MPa or more, cause cell death in this processing system; however, the conditions of the system have to be adapted for the different food groups to be processed, so it is recommended that for each type of new product, parameters such as pressurization time and temperature, presence of one or more antimicrobial agents, pH and water activity are evaluated. In pressurization, pressure rise and fall times generally vary from 1 to 5 minutes, depending on the equipment. During processing, the pressure compresses the food from 10 to 15%, in the range of 300 to 700 MPa, and raises the temperature by adiabatic heating to an approximate ratio of 3°C for every 100 MPa of pressure applied in the system. These changes are temporary and are restored in depressurization to the original states. In this process both the increase and decrease of pressure directly influence the microbial deactivation, causing the phase transition of the lipids of the bacterial membrane, and the rupture of the ionic bonds, hydrophobic interactions and formation of hydrogen bridges, between the molecules, without affecting the covalent bonds, resulting in the unfolding of the macromolecules as proteins. This negatively affects other structural and functional components of microbial cells, losing viability. Since pressurization does not act on the covalent bonds of macromolecules, it does not affect vitamins, pigments or other molecules, favoring their use in the conservation of these nutrients, in comparison with conventional thermal treatments. It is considered that damage to the cell membrane is the main cause of cell death, however, other possible causes reported are damage to the cell wall and nucleic acids.

Xie *et al* [7] studied the structural characteristics, physicochemical properties and morphological characteristics of pectin treated with high hydrostatic pressure and high-pressure homogenization. The authors suggest that this high-pressure processing is an efficient technique for modifying the pectin from potato peel wastes to a thickening or stabilizing agent, but high-pressure homogenization shows a better effect. Another study was aimed at optimizing the individual and interactive effect of high pressure operation and polarity of solvents (solvent mix) on extraction yield, flavonoid and lycopene content of tomato pulp. The authors concluded that high hydrostatic pressure could be a useful tool to improve the extraction and release of compounds potentially beneficial to health [8]. Further, high hydrostatic pressure treatment is used as a new food preservation technique because of its ability to inactivate pathogenic and spoilage bacteria and minimize food quality loss. This study therefore reviews the effects of sublethal (≤ 100 MPa) and lethal (N100 MPa) pressures on protein synthesis, structure and functionality in bacteria. Most notably, high hydrostatic pressure, in conjunction with certain enzymatic reactions, may have great potential for biotechnological applications [9].

#### 2.1.2. Dielectric heating

The dielectric heating has been applied as a methodology to conservation and improve the quality of food products. This technique uses a range of 1–300 MHz, particularly 13.56, 27.12 and 40.68 MHz when used for commercial applications. The radiofrequency heating offers significant advantages such as faster, better quality, more uniform heat distribution and higher energy efficiency for solid and semi-solid foods with a low thermal conductivity as compared to other conventional treatment methods [10]. For example, wheat germ is a valuable by-product of wheat milling but is highly susceptible to lipid rancidity induced by lipase activity. In this study show that dielectric constant and dielectric loss factor of wheat germ increased with increasing temperature and moisture content. Overall, the results obtained by Ling *et al* [11]. are useful in computer simulation and optimizing process parameters for wheat germ stabilization by radiofrequency heating. On the other hand, non-uniform heating is a major challenge for using radiofrequency heat treatment in pasteurization of low moisture food products. This study provided useful information to develop an effective radiofrequency process as an alternative to conventional thermal treatments for pasteurization of low moisture products. Therefore, it is a novel methodology based on radio frequency heating to pasteurize food powder [12].

#### 2.1.3. Pulsed light

Pulsed light is a photonic technology mainly studied for microbial inactivation, especially in the food technology field. It consists of the application of pulses of high-intensity broad-spectrum light that includes UV light. The main feature of pulsed light technology is the production of high photon-fluxes [13]. For example, *Cronobacter sakazakii* and *Salmonella* spp. are foodborne pathogens associated with low moisture foods. Chen *et al* [14] developed an intense pulsed light system as an alternative novel method to pasteurize powdered food. The aim of this study was to investigate the microorganism inactivation in different powdered foods and a variety of related variables using a vibratory-assisted intense pulsed light system. The authors concluded that the enhanced microbiological inactivation could be achieved using this vibratory-assisted intense pulsed light system after multiple passes. In another study, intense pulsed light treatment at a total fluence of 7.40 J/cm^2^ resulted in a 7 log reduction, indicating the potential of intense pulsed light to inactivate bacterial spores effectively. The best results showed that the inactivation efficiency increases after one h pre-incubation because the numbers of vegetative cells increased with the incubation time [15].

#### 2.1.4. Bacteriocins

Bacteriocins are peptide substances with antimicrobial and bioconservative activity that are produced by different strains and play an important role in food preservation [16]. They can be used in a wide range of food systems and are synthesized ribosomal and extracellularly. A large number of bacteriocins have been isolated and characterized; however, food biopreservation has mostly focused on bacteriocins of lactic acid bacteria [17]. The most important are nysin, diplococine, acidophiline, bulgarican, helveticine, lactaine and plantaricin [18]. It can be used in meat, dairy products, canned products, seafood, vegetables, fruit juices and beverages such as beer and wine. Its compatibility characteristics in these products, as well as its mode of action make its use in food attractive.

In recent years, pure starter cultures with bacteriocin-producing capacity have been applied to the manufacture of safer fermented fish products. Currently, most of the meat with commercially used probiotic bacteria belongs to the genus *Lactobacillus*, including *Lb. casei, Lb paracasei, Lb johnsonii, Lb plantarum* and *Lb rhamnosus* [19,20]. Nisin is produced by different *Lactococcus lactis* spp. and is the most studied bacteriocin to date and the only bacteriocin applied as a food additive worldwide. Salvucci *et al* [21] obtained active triticale flour films by adding bacteriocin-like substances (BLIS) produced by *Enterococcus faecium* ES216 with antimicrobial activity against *Listeria innocua* ATCC33090. The authors suggest that triticale flour films activated with these bacteriocins could provide an alternative for active food packaging applications. In another study, a strain of *Lysinibacillus* isolated from spoiled fruits and vegetable wastes was found having an inhibitory effect against foodborne pathogens. Subjected to the study of different physicochemical parameters followed by characterization based on the 16S rRNA, a novel bacteriocin of class III was found. This bacteriocin was highly active against the foodborne pathogen *Bacillus pumilus* (MIC-22 μg/mL). The authors suggested that *Lysinibacillus* JX402121 isolate can be utilized in the production of bacteriocin that acts as a bio-preservative agent against different food-borne pathogens [22]. Due to their protein nature, bacteriocins are inactivated by proteases, including those of pancreatic and gastric origin, due to this during their passage through the gastrointestinal tract are inactivated, without being absorbed as active compounds and thus presumably harmless to the consumer [23].

### 2.2. Extractive emerging technologies & biomolecules

In recent years, the development of the food industry has focused on generating products that not only satisfy the need for food but also meet consumer demand for nutrient-enhanced foods, mainly those enriched with antioxidants and/or compounds that promote some beneficial effect on health. The supplementation of food has been given by the incorporation of compounds that are extracted through various techniques.

Green technologies have been developed as an alternative to conventional extraction technologies because they allow for the recovery of a greater quantity of bioactive compounds of interest, use shorter extraction periods (hours of reduction to seconds), and increase the quality of extracts with lower processing costs, or the extraction of compounds that are difficult to obtain through conventional techniques [24,25]. Some of these techniques are: ultrasound-assisted extraction, ohmic heating, high pressure, supercritical fluid extraction and microwave-assisted extraction [26–29]. Green techniques have the advantage of being more efficient and environmentally friendly because these techniques use less solvents and energy, consume less extraction time and are simpler compared to conventional techniques, and there is less degradation of thermolabile compounds, better products and higher yields [27–29].

#### 2.2.1. Microwave-assisted extraction (MAE)

Extraction through microwave is based on the incidence of microwave waves that generate an increase in temperature because the energy of the waves generates vibrations in the molecules contained in the medium which in turn translates into increased temperature [30,31]. The vibrations interfere with the cell membranes generating a disruption of them and consequently the cell content is poured causing the release of antioxidant compounds and/or intracellular bioactive compounds [32,33] (Table 1). This methodology is characterized by the high yields reported, as well as the decrease in the number of solvents used and the extraction times of the bioactive compounds [34,35]. Also, it uses 95% of the energy supplied, thus reducing environmental pollution with shorter extraction times and lower CO_2_ emissions to the atmosphere [28,36].

**Table 1.** Methods of extraction of bioactive compounds that are used in the food industry by emerging technologies.

Method of extraction	Conditions	Recovered bioactive compounds	References
Ultrasound assisted extraction	Extraction time of 25 min Temperature of 45°C Ultrasound amplitude 47% Solvent 80% methanol	Phenolic compounds	[34]
	Extraction time of 20 min, Temperature of 62 °C Ultrasound power of 404 W.	Polysaccharides	[37]
	Extraction time of 20 min Ultrasound power of 20 kHz	Pigments (Astaxanthin)	[38]
	Extraction time 15–30 min Temperature of 50 °C Ultrasound power of 150 W.	Polysaccharides (alginates and carrageenans)	[52]
Microwave assisted extraction	Temperature of 36°C Microwave power of 800 W	Anthocyanins	[33]
	Extraction time of 14min Microwave power of 600W	Essential oil	[39]
	Extraction time 2.5 min Microwave power of 517 W	Pectin	[40]
	Extraction time 20 min Temperature 100°C Solvent Ethanol (50%)	Polyphenols	[41]
Supercritical Fluid Extraction	Extraction time 76 min Temperature 50°C	Amino acids	[42]
	Temperature 53.2°C Pressure 25.54 MPa	Essential oil	[43]
	Temperature of 68°C Pressure of 205 bar Co-Solvent Ethanol (15.5%)	Polyphenols	[44]
	Temperature of 45°C Pressure of 225 bar	Steviol glycosides and phenolic compounds	[45]

Microwave irradiation generates electromagnetic radiation with wavelengths between 1mm and 1m. In the electromagnetic spectrum is between 300 and 300,000 MHz, this non-ionizing radiation selectively transfers energy in various substances [46]. MAE has been shown to generate more advantages compared to conventional heating such as: fast heat transfer in a short reaction time, uniform volumetric heating, energy efficiency, low level of degradation or formation of secondary products [47]. The difference between conventional heating and microwave heating is observed in heat transfer, since in conventional heating the energy is transferred from the outer surface of the fibres to the inside of the core by means of conduction, so that overheating occurs in the upper part while the inner part remains cold, whereas the use of the microwave technique the heat is induced at the molecular level by direct convection, so that the energy is distributed throughout the material [46]. The effect of heat on plant fibres is related to reacting with the molecules that cause ionic conduction and rotation of dipoles [48], where an electric field is generated by the transfer of ions and electrons from the microwave, this ionic conduction generates a movement of particles, where the displacement of polar molecules is based on the rotation of dipoles. This movement is caused by the effort to align to the existing electric field, generating a release of energy and a fast and uniform heating. This uniformity promotes an internal diffusion causing a fast damage to the cells of the material as well as the diffusion of the compounds, for which the cellular walls are broken by means of the expansion, for the later penetration of the solvent and the diffusion of the desired compounds out of the cellular matter [49].

#### 2.2.2. Ultrasound-assisted extraction (UAE)

The ultrasound is in the range of 20 KHz – 1MHz and this extractive methodology is one of the most adopted today; ultrasonic waves allow the generation of a process called gas cavitation which consists of the formation of microbubbles in a liquid medium [50,51]. The cavitation process generates cell breakage which produces the entry of solvent into the cell and an intensification of mass transfer, promoting the release of the bioactive compounds sought [52]. This methodology is widely used in the food industry for chemical processing, food processing, drying, emulsification, homogenization, the inactivation of enzymes and pathogenic microorganisms, sanitation of surfaces and equipment, as well as the removal of gases in liquids [34] (Table 1).

This technique used in vegetable sources produces an alteration of the surface, in addition free radicals are generated that alter the lignocellulosic matrix [53], which generates the rupture of links in the lignin causing a division of the polysaccharides that conform it [54]. UAE also offers advantages over other conventional techniques such as shorter exposure time, less use of solvents, higher yields, high reproducibility and uses little energy [55]. This technology is recommended for samples containing volatile and semi-volatile organic compounds as it can be performed at room temperature and normal pressure.

#### 2.2.3. Super critical fluid extraction (SCE)

The extraction of bioactives through supercritical fluids has taken a great interest, as they have many advantages, especially due to the high extraction efficiency [56,57]. A supercritical fluid is a substance that is under operating conditions of pressure and temperature higher than its thermodynamic critical point. At the critical point, the properties of the liquid and gas phase become similar as to be indistinguishable, and the solvents that are in their critical point are characterized by high diffusivity. Supercritical fluid has the property of diffusing through solids as a gas and dissolving materials as a liquid [58,59]. These properties make it suitable as a substitute for organic solvents in the extraction processes. Carbon dioxide is the most used because it is an ideal solvent to be non-explosive, nontoxic, the requirements can be met in addition to its removal from the extracts is easy and fast [60,61]. Another advantage of this fluid is that the critical conditions are reached at low temperatures which allow thermolabile compounds to be separated (Table 1). Extraction with supercritical fluids is considered a green extractive technology [62]. High pressures (100–800 MPa) can easily interrupt electrostatic and hydrophobic interactions between molecules and increase mass transfer and cell permeability rate, leading to increased release of compounds with functional properties. This method is very efficient for the extraction of nonpolar compounds, but some solvents such as ethanol and water could be used to increase the solubility of polar compounds. The extraction of antioxidant compounds and other bioactive compounds from plants, fruits and agro-industrial residues through the supercritical fluid method has been reported in recent years.

### 2.3. Develepment of biomaterials & bioproducts

The development and application of biomass derivate materials, so-called biomaterials, has steadily increased over the last years. This is mainly due to abundant use of low-cost agricultural byproducts and coproducts and their ability to improve the development of products that lead to the generation of new materials whose applicability in the industry represents a real technological advantage. The development of bioproducts is a step forward toward ensuring that fossil resources are replaced by sustainable natural alternatives for the production of new highly promising materials in the food industry. A biomaterial can be a material complete or partly produced from a renewable resource. There is a variety of raw materials suitable for development for biomaterials, such as soy, wood fibres, corn, and residues of harvesting food crops.

#### 2.3.1. Biopolymers

Recently, the research in new biomaterials and bioprocesses has directed to the development of engineering focused on obtaining polymeric materials with application in the food industry. The vegetable matter with abundant content of starches, sugars and oils, represent a source of significant raw material since from its use has been achieved the development of biotechnological processes that allow obtaining natural polymers with plastic properties [63]. Biopolymers can be synthetized from different natural sources: polysaccharides, proteins, wood, vegetable waste materials, polylactic acid (synthesized from renewable biobased monomers), natural oils and polyhydroxyalkanoates represents a potential source of renewable feedstocks [64,65]. Biopolymers represent, in many cases, the development of materials of high industrial value. The development and use of these materials has led to the practice of bioengineering to explore relationships between processes, structures and functions in food products, designing materials and processes that impart greater safety, improve sensorial properties, cost, efficiency and sustainability. Among the biofilm options, we can mention recent developments that represent great potential in the food industry. Development of bioplastic fibres from gum arabic have shown significant antibacterial activity, low oxygen barrier properties, potent antioxidant activity and promising biodegradability [64]. Chitosan can be used to generate polymeric films with valuable functional properties. Mixtures of chitosan with wood hydrolyzate were prepared and used to form films and coatings by application on PET film showing suitable mechanical properties and very low oxygen permeabilities [66]. Chitosan-based films combined with biopolymers, like polysaccharides, proteins, extracts or organic acids have been developed with improved properties and characteristics, like antibacterial, barrier and sensing properties, showing great potential for food packaging applications [67].

#### 2.3.2. Bioactive packaging

The develop of functional technology, also known as bioactive packaging, has a straight influence on the nutritional benefits of a food product by generating healthier packaged foods. The progress in the development of biodegradable and sustainable matrixes have led to the integration of bioactive substances, that in combination with biopolymers are able to provide properties that prolongs the storage life and enhances the margin of food safety by altering the condition of the food allowing to improve its conservation [68]. Bioactive packaging materials would thus be capable of withholding desired bioactive principles in optimum conditions until their eventual release into the food. Innovative functional packaging concepts are devised to give answer to the current barriers and limitations in the manufacturing functional foods [68]. The potential of these technologies also depends on the techniques to implement it. The use of biodegradable packaging allows the diffusion of bioactive components, through encapsulation of the bioactive ingredients in the packaging material. These materials can also be used as biodegradable materials with enzymatic activity with the ability to transform the food components in order to provide food quality, reduce food waste and increase its shelf life [69,70]. The advancement in the use of bioactive components in packaging films is the addition of additives. Guo *et al* [63] demonstrated the antimicrobial activity of chitosan-polylactic acid packaging films in ready-to-eat meat products. These films, containing multiple acid solutions in presence of lauric arginate ester, sodium lactate and sorbic acid significantly inhibited the growth of *Listeria* and *Salmonella* microorganisms on during storage. Ounkaew *et al* [71] used polyvinyl alcohol-starch films prepared by incorporating combined antioxidant agents, improving the efficiency of the antimicrobial packaging films.

## 3. Bioprocess design

As mentioned above, consumers are increasingly demanding foods that are added to/fortified with ingredients that promote a beneficial effect. For this reason, obtaining and recovering high value-added compounds in the food industry has become an increasingly important activity. These compounds include amino acids, enzymes, organic acids, vitamins, antibiotics, gums, oligosaccharides (fructo-, malto- and xylo-, oligosaccharides), among others. Production of chemicals via fermentation has several advantages when compared to chemical syntheses, such as low-cost substrates, relatively lower temperatures, lower energy consumption, better environmental concerns, high purity plus ease of creating products with tailor-made characteristics [72]. Optimization of these processes can be achieved by bioprocess design.

Process design is the conceptual work done prior to building, expanding or retrofitting a process plant. It consists of two main activities, process synthesis and process analysis. The synthesis is the selection and arrangement of a set of unit operations capable of producing the desired product at an acceptable cost and quality [73]. Process analysis is the evaluation and comparison of different process synthesis solutions. The process is divided into two phases: upstream and downstream processing accounts for a large proportion of the production costs [72,73].

The vast majority of important decisions for capital expenditures and product commercialization are based on results of preliminary process design and cost analysis [74]. In some cases, implementation of bioprocess analysis, from genetic engineering, fermentation and downstream processing to final application testing is key to develop new strains and processes [75].

Bioprocess design is used in virtually every biological process, from surfactants and recombinant protein production to human stem cell culture [75–77].

### 3.1. Operational fermentation strategies

There are a vast number of strategies and techniques to recover products from fermentation. Generally, they can be grouped in 1) removal of most plentiful impurities; 2) Removal of the easiest-to-remove impurities; 3) Selection of processes that take advantage of the differences of the product and the impurities.

However, some of the first steps are establishing differences between intra and extracellular products being simpler the recovery of the last one. The steps for the recovery of these products are mentioned ahead. The operations depend on the characteristics of the final product. Usually, batch culture is used for high-value products and continuous production and separation are used for the production of commodity biochemicals [73].

Initial stages comprehend the purification and concentration of the work volume. Ahead, we mention some of the most common strategies for these purposes for intracellular products and later on, for extracellular ones.

### 3.2. Intracellular products

Cell harvest. In these steps, the most impurities are eliminated. Most common techniques for this purpose are membrane filtration and centrifugation. Cellular disruption. Usually, this is the second step and it disrupts the cells to release the product. These can be done using mill beads, high-pressure homogenizer and osmotic shock. For this stage is necessary to have a ‘lysis buffer’ suitable for minimizing product denaturation [72,74] Cell debris removal. This debris is generated by cell disruption and is usually removed by centrifugation or microfiltration. Other options are rotary vacuum filtration, press filtration, depth filtration, extraction and expanded bed adsorption chromatography [72]. Soluble product. When the product is soluble, it is recovered during cell debris removal in the supernatant. Usually, a polishing step is needed to remove small debris particles. Filters can be used for this purpose. Two aqueous phase extraction can be applied for this purpose [78]. Insoluble product. When the product forms inclusion bodies, it must be separated from the cell debris, then dissolved and refolded. Fortunately, inclusion bodies are large and dense and can be separated by centrifugation. Product extraction/adsorption. Product separation can be carried out by extraction and/or adsorption [74].

### 3.3. Extracellular products

Biomass removal. Usually, biomass is the easiest-to-remove impurities and it can be removed by decantation, filtration, centrifugation, etc. Intermediate Recovery Stages. These stages are where the product is concentrated and further purified. Here, the product is concentrated and renatured to move onto the final purification stages. These depend on the required final product purity. Pharmaceutical products require high purity while industrial products require lower purity. Chromatography, two aqueous phases extraction are some popular techniques [76,78].

### 3.4. Process analysis

Flowsheets of the process must be analyzed and compare don the basis of capital investment, manufacturing cost, environmental impact and other criteria in order to make decisions. There are tools for these tasks such as spreadsheets, process simulators that allow making flowsheets of the process [73]. The preliminary economic evaluation of a Project for manufacturing a biological product usually involves the estimation of capital investment, estimation of operating costs and analysis of profitability. The results of these analyzes provide information for every step of the bioprocess design and could be used to improve the efficiency of the process, from raw materials, type of bioreactor and the downstream process [72].

**Figure 1.**
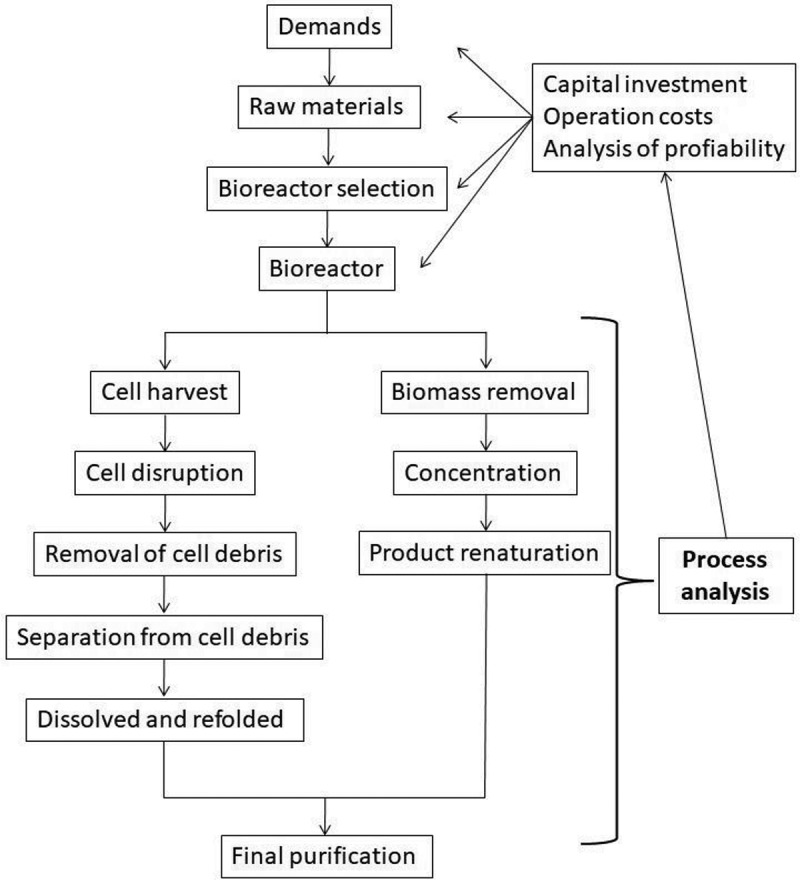
Flow sheet for bioprocess design.

A flowsheet for bioprocess design is provided ahead in Figure 1.

## 4. Other bio-based tools

### 4.1. Synthetic biology

Synthetic biology is the biomolecular synthesis or biological systems engineering that synthesizes complex systems based on biology, and that produces new functions that are not found in nature, whether in organisms, food or for therapies [79]. The main difference with traditional genetic engineering is that existing genomes are not modified but provides organisms with genotypic characteristics of industrial interest with complex systems that can be built and designed, which show biological functions of organisms that do not exist in nature [80,81].

The term synthetic biology was coined to describe a bacterium that had been genetically engineered using recombinant DNA technology. These bacteria are living systems that have been altered by human intervention (synthetically) [82]. Today, that technology has allowed researchers to do not only manipulate genes and analyze genomes; they can also build a life from scratch. In this way, natural organisms with little complexity are used and functions required when designing them in the existing genome are added. Some of these modifications that are designed are genomes modification, production and analysis of artificial cells, designing cellular components, pathway engineering and the creation of synthetic biomolecules with some specific function [79,82]. Today, synthetic biology is widely in the food industry through the development of enzymes, tissue and microorganisms that improve their conservation, flavor and nutritional activity [83,84].

### 4.2. Impact and applications of synthetic biology in the food industry

One of the main concerns of food companies is to take care of the quality and biosecurity standards of their products, as well as the quality and nutrition systems of their products. Implementing efficient, environmentally friendly, sustainable and high innovation processes. It is here that synthetic biology has been used, becoming an essential and innovative tool in the food, dairy and agriculture industry [85].

This technology has allowed the development of foods with greater and better nutritional properties, including some made for each specific need of the consumer as allergies or intolerances of some foods. Also, it has allowed the design and production of food with modifications of color, taste, smell, vitamin content, fibre, protein, fat and carbohydrates. In addition, designs of food methods rich in caloric content and fat, among others, have been made to diminish them [86,87].

Likewise, synthetic biology can also be used to create food packaging and biofilms to maintain shelf life and preserve the properties of food (flavor, color, smell and nutrients) for longer, even keeping them free of microorganisms that affect them or pollute [88]. On the other hand, this technology is also used to produce fertilizers, plant development treatments and pesticides, due to the use of enzymes and molecules produced exclusively to generate that benefit to agriculture. Also favoring the manufacturing processes of the food industry and food safety systems in the cultivation fields through various molecular techniques to treat diseases and produce biosensors for the detection of pathogens [89,90,91].

Moreover, synthetic biology has been used like the advantage of natural biodegradative pathways in certain microorganisms to remediate potent environmental contaminants such heavy metals, actinides and nerve agents. Another approach to control and eliminate pathogenic microorganisms in cultures is the use of bacteriophages produced by synthetic biology. These viruses are harmless to humans, animals, and plants because they specifically target and kill microorganisms of interest. These processes confer antimicrobial activity because the virulent phages follow a lytic cycle where they multiply within bacteria to finally induced lysis of the cell [92].

For all the above, synthetic biology has become a technology of great importance and high impact in the food industry, agriculture, medicine and health. It encompasses various sciences such as biotechnology, engineering, molecular, cellular and nutrition biology, used throughout the scientific world. Therefore, it is considered a highly recommended technology as a strategy for the development of industries.

### 4.3. Bioinformatics in the food industry

In the last decade, Next-Generation Sequencing (NGS) has become a routinely applied tool in many fields, including the food industry, especially in food microbiology. This technology can be used either to determine the whole genome of a single cultured isolate and thus be referred as ‘Whole Genome Sequencing’ (WGS) or to generate sequences of multiple microorganisms in a biological sample, which is known as metagenomics. These technologies are replacing traditional microbial typing and characterization techniques such as serotyping, antimicrobial resistance determination and virulence profiling due to faster and more precise results, and even higher discriminatory power than traditional molecular typing [93]. WGS technology has been introduced for surveillance of foodborne pathogens in the United Kingdom, France, Denmark, and The United States, allowing the detection of more and smaller outbreaks, identification of the outbreaks source and related cases of listeriosis [94]. On the other hand, metagenomics has been used to predict the presence or emergence of spoilage and pathogens microorganisms as well as to characterize unknown microbiota, thus being useful for food safety and quality improvement.

NGS comprises both massively parallel and single-molecule sequencing, which provides short (100–300 pb) and long (10 to 50 Kb) sequencing reads. A large amount of data generated through this technology has led to the development of multiple software solutions and bioinformatics specialty. The approaches to analyze these genomic data in combination with epidemiological evidence enhances the ability to determine the source of infection, transmission route [95], or relatedness between strains. It can be useful also to measure evolution within bacteria or to produce phylogenetic trees by the changes in their DNA [96]. Phylogeny reflects epidemiological relatedness: food or environmental isolates that are phylogenetically closely related are likely to be epidemiologically or causally linked [97]. However, food traceback and epidemiological evidence must be to support the correct interpretation.

Even standardization of techniques and well-curated and high-quality database of genomic sequence for pathogenic, functional microbes and probiotics is needed for implementation of NGS methods for food safety management [98], groups such as the Consortium for Sequencing the Food Supply Chain (CSFSC) are putting efforts into characterizing and quantifying the microbiome before and after processing as well as collecting genome information on pathogenic bacteria across the food supply chain to assure food safety, traceability and authenticity [99].

Metagenomics, coupled with new bioinformatic tools, has become a powerful strategy in diagnostics, monitoring, and traceability of products also. In an example, its recent application in viticulture is promising, where enology direction is emerging to blend the industrial safety of monitored fermentation with spontaneous fermentation due to the great acceptance among consumers of wines with distinctive autochthonous peculiarities. However, it implies that population dynamics within the entire winemaking process must be understood, as well as their ecological niches, the relationship between microbiome-wine health and microbiome-wine metabolome, their biological roles and the technical basis [100].

Bioinformatics can also be used in combination with genetic recombination technology to develop microbial bioproduct advancements to benefic industrial production un a cost-effective way. This was the case of Meng *et al* [101], who developed a recombinant *Bacillus subtilis* for the production of pullulanase using different promoters to improve yield. This enzyme is of especial interest in the industrial starch fermentation for the production of alcohols, amino acids, high-glucose and high-maltose syrups, which relies on pullulanase to degrade a-1,6-glycosidic linkages to improve starch hydrolysis [102]. Elaziz *et al* [103] also used bioinformatics to generate a prediction algorithm to enhance the properties of fish products with therapeutic and industrial roles, bioactive amino acids. Using biotechnological approaches, the concentration level of these compounds was assessed in protein hydrolyzates extracted from tilapia fish.

## 5. Conclusions

This review highlighted the relevance, advantages and current improvement in plant, animal and microbes bioengineered tools and outlines feasible approaches by biological and process’s bioengineering levels for advancing the economic feasibility of food industries. The critical analysis results revealed that the bioengineering tools and strategies could be of promising impact on the development of the food industry. Methods to overcome and resolve the accompanying encounters of food’s production. As a conclusion, it also gives new insight into the challenges and possible breakthrough of the development of food bioproducts and new ingredients through bioengineered tools

## Highlights

- Emerging and Bioengineered tools and strategies for the development of the food industry;
- Integration of bioprocess, bioproducts, synthetic biology and bioinformatical approaches are discussed;
- Critically described the challenges and future possibilities for the bioengineered tools for food industry.

## References

[1] Morata Barrado A. New Food Consevation Technologies. Vol. 2 Madrid, España: A. Madrid Vicente Ediciones; 2010.

[2] Knorr D Effects of high-hydrostatic-pressure processes on food safety and quality. Food Technol. 1993;47(6):156–161.

[3] Hoover DG Minimally processed fruits and vegetables: reducing microbial load by non-thermal physical treatments. Food Technol. 1997;51(6):66–71.

[4] Meyer RS, Cooper KL, Knorr D, et al High pressure sterilization of foods. Food Technol. 2000;54(11):67–71.

[5] Cheftel JC Review: high-pressure, microbial inactivation and food preservation. Food Sci Technol Int. 1995;1:75–90.

[6] Kimura K, Ida M, Yosida Y, et al Comparison of keeping quality between pressure-processed jam and heat-processed jam: changes in flavor components, hue, and nutrients during storage. Biosci Biotech Biochem. 1994;58(8):1386–1391.

[7] Xie F, Zhang W, Lan X, et al Effects of high hydrostatic pressure and high pressure homogenization processing on characteristics of potato peel waste pectin. Carbohydr Polym. 2018;196:474–482. 29891321 10.1016/j.carbpol.2018.05.061

[8] Briones-Labarca V, Giovagnoli-Vicuña C, Cañas-Sarazúa R Optimization of extraction yield, flavonoids and lycopene from tomato pulp by high hydrostatic pressure-assisted extraction. Food Chem. 2019;278:751–759. 30583438 10.1016/j.foodchem.2018.11.106

[9] Gayán E, Govers S, Aertsen A Impact of high hydrostatic pressure on bacterial proteostasis. Biophys Chem. 2017;231:3–9. 28365058 10.1016/j.bpc.2017.03.005

[10] Ozturk S, Kong F, Singh R, et al Dielectric properties, heating rate, and heating uniformity of various seasoning spices and their mixtures with radio frequency heating. J Food Eng. 2018;228:128–141.

[11] Ling B, Lyng J, Wnag S Radio-frequency treatment for stabilization of wheat germ: dielectric properties and heating uniformity. Innovative Food Sci Emerging Technol. 2018;48:66–67.

[12] Ozturk S, Kong F, Singh R, et al Radio frequency heating of corn flour: heating rate and uniformity. Innovative Food Sci Emerging Technol. 2017;44:191–201.

[13] Martínez-López S, Lucas-Abellán C, Serrano-Martínez A, et al Pulsed light for a cleaner dyeing industry: azo dye degradation by an advanced oxidation process driven by pulsed light. J Clean Prod. 2019;217:757–766.

[14] Chen D, Chen Y, Peng P, et al E?ects of intense pulsed light on Cronobacter sakazakii and Salmonella surrogate Enterococcus faecium inoculated in di?erent powdered foods. Food Chem. 2019;296:23–28. 31202302 10.1016/j.foodchem.2019.05.180

[15] Jo H, Hwang H, Chung M Inactivation of Bacillus subtilis spores at various germination and outgrowth stages using intense pulsed light. Food Microbiol. 2019;82:409–415. 31027800 10.1016/j.fm.2019.03.013

[16] Settanni L, Corsetti A Application of bacteriocins in vegetable food biopreservation. Int J Food Microbiol. 2008;121(2):123–138. 18022269 10.1016/j.ijfoodmicro.2007.09.001

[17] García P, Rodríguez L, Rodríguez A, et al Food biopreservation: promising strategies using bacteriocins, bacteriophages and endolysins. Trends Food SciTechnol. 2010;21:373–382.

[18] Savadogo A, Ouattara C, Bassole I, et al Bacteriocins and lactic acid bacterias - a minireview. J Biotechnol. 2006;5(9):678–683.

[19] Wang Y, Sun Y, Zhang X, et al Bacteriocin-producing probiotics enhance the safety and functionality of sturgeon sausage. Food Control. 2015;50:729–735. Elsevier Ltd.

[20] Avaiyarasi ND, AD Ravindran, Venkatesh P, et al In vitro selection, characterization and cytotoxic effect of bacteriocin of Lactobacillus sakei GM3 isolated from goat milk. Food Control. 2016;69:124–133. Elsevier Ltd.

[21] Salvucci E, Rossi M, Colombo A, et al Triticale ?our ?lms added with bacteriocin-like substance (BLIS) for active food packaging applications. Food Pack Shelf Life. 2019;19:193–199.

[22] Ahmad V, Ahmad K, Baig M, et al Efficacy of a novel bacteriocin isolated from Lysinibacillus sp. against Bacillus pumilus. LWT - Food Sc Technol. 2019;102:260–267.

[23] Quintero J, Falguera V, Muñoz A Películas y recubrimientos comestibles?: importancia y tendencias recientes en la cadena hortofrutícola. Revista Tumbaga. 2010;5:93–118.

[24] Martins S, Aguilar CN, De la Garza I, et al Kinetic study of nordihydroguaiaretic acid recovery from Larrea tridentata by microwave-assisted extraction. J Chem Technol Biotechnol. 2010;85:1142–1147.

[25] Yan MM, Liu W, Fu YJ, et al Optimisation of the microwave-assisted extraction process for four main astragalosides in Radix Astragali. Food Chem. 2010;119:1663–1670.

[26] Xia E, Ai X, Zang S, et al Ultrasound-assisted extraction of phillyrin from Forsythia suspensa. Ultrason Sonochem. 2011;18:549–552. 20980187 10.1016/j.ultsonch.2010.09.015

[27] Chan CH, Yusoff R, Ngoh G, et al Microwave-assisted extractions of active ingredients from plants. J Chromatogr A. 2011;1218(37):6213–6225. 21820119 10.1016/j.chroma.2011.07.040

[28] Perino S, Huma Z, Abert M, et al Solvent Free Microwave-Assisted Extraction of Antioxidants from Sea Buckthorn (Hippophae rhamnoides) Food By-Products. Food Bioprocess Technol. 2011;4:1020–1028.

[29] Taamalli A, Arráez D, Ibañez E, et al Optimization of Microwave-Assisted Extraction for the Characterization of Olive Leaf Phenolic Compounds by Using HPLC-ESI-TOF-MS/IT-MS2. J Agric Food Chem. 2012;60:791–798. 22206342 10.1021/jf204233u

[30] Kumar C, Benal MM, Prasad BD, et al Microwave assisted extraction of oil from pongamiapinnata seeds. Mater Today Proc. 2018;5:2960–2964.

[31] Afolabi HK, Mudalip SKA, Alara OR Microwave-assisted extraction and characterization of fatty acids from eel fish (Monopterus albus). Beni-Suef University. J Basic Appl Sci. 2018;7:465–470.

[32] Cassol L, Rodrigues E, Noreña CPZ Extracting phenolic compounds from Hibiscus sabdariffa L. calyx using microwave extraction. Industrial Crops & Products. 2019;133:168–177.

[33] Liu C, Xue H, Shen L, et al Improvement of anthocyanins rate of blueberry powder under variable power of microwave extraction. Sep Purif Technol. 2019;226:286–298.

[34] Setyaningsih W, Saputro IE, Carrera CA, et al Optimisation of an ultrasound-assisted extraction method for the simultaneous determination of phenolics in rice grains. Food Chem. 2019;288:221–227. 30902286 10.1016/j.foodchem.2019.02.107

[35] Pimentel-Moral S, Borrás-Linares I, Lozano-Sánchez J, et al Microwave-assisted extraction for Hibiscus sabdariffa bioactive compounds. J Pharm Biomed Anal. 2018;156: 313–322. Nutrition Reports International 37: 1329–1337. 29734100 10.1016/j.jpba.2018.04.050

[36] Yemis O, Mazza G Optimization of furfural and 5-hydroxymethylfurfural production from wheat straw by a microwave-assisted process. Bioresour Technol. 2012;109:215–223. 22297050 10.1016/j.biortech.2012.01.031

[37] You L, Bin P, Yan L, et al Ultrasound extraction of polysaccharides from guava leaves and their antioxidant and antiglycation activity. Process Biochem. 2018;73:228–234.

[38] Batghare AH, Pati S, Roy K, et al Mechanistic investigations in ultrasound-assisted extraction of astaxanthin from Phaffia rhodozyma MTCC 7536. Bioresour Technol Rep. 2018;4:166–173. 10.1016/j.biortech.2018.01.073 29413919

[39] Chandra Kumar R, Bernal MM, Durga Prasad B, et al Microwave assisted extraction of oil from pongamiapinnata seeds. Mater Today Proc. 2018;5:2960–2964.

[40] Lefish K, Giacomazza D, Dahmoune F, et al Pectin from Opuntia ficusindica: optimization of microwave-assited extraction and preliminary characterization. Food Chem. 2017;221:91–99. 27979293 10.1016/j.foodchem.2016.10.073

[41] Moreira MM, Barroso MF, Boeykens A, et al Valorization of apple tree wood residues by polyphenols extraction: comparision between conventional and microwave-assisted extraction. Industrial Crops & Products. 2017;104:210–220.

[42] Varaee M, Honarvar M, Eikani MH, et al Supercritical fluid extraction of free amino acids from sugar beet and sugar cane molasses. J Supercrit Fluids. 2019;144:48–55.

[43] Priyanka SK Influence of operating parameters on supercritical fluid extraction of essential oil from turmeric root. J Clean Prod. 2018;188:816–824.

[44] Krakowska A, Rafinska K, Walczak J, et al Enzyme-assisted optimized supercritical fluid extraction to improve Medicago sativa polyphenolics isolation. Industrial Crops & Products. 2018;124:931–940.

[45] Ameer K, Chun BS, Kwon JH Optimization of supercritical fluid extraction of steviol glycosides and total phenolic content from Stevia rebaudiana (Bertoni) leaves using response surface methodology and artificial neural network modeling. Industrial Crops & Products. 2017;109:672–685.

[46] Huang Y-F, Chiueh P-T, Kuan W-H, et al Microwave pyrolysis of lignocellulosic biomass: heating performance and reaction kinetics. Energy. 2016;100:137–144.

[47] Dai L, He C, Wang Y, et al Comparative study on microwave and conventional hydrothermal pretreatment of bamboo sawdust: hydrochar properties and its pyrolysis behaviors. Energy Convers Manag. 2017;146:1–7.

[48] Wang H, Ding J, Ren N Recent advances in microwave-assisted extraction of trace organic pollutants from food and environmental samples. Trends Analyt Chem. 2016;75:197–208.

[49] Carbonell-Capella JM, Šic Žlabur J, Rimac Brncic S, et al Electrotechnologies, microwaves, and ultrasounds combined with binary mixtures of ethanol and water to extract steviol glycosides and antioxidant compounds from Stevia rebaudiana leaves. J Food Process Preserv. 2017;41(5):e13179.

[50] Trojanowska A, Tsibranska I, Dzhnonova D, et al Ultrasound-assisted extraction of biologically active compounds and their successive concentration by using membrane processes. Chem Eng Res Des. 2019;147:378–389.

[51] Preece KE, Hooshyar N, Krijgsman A, et al Intensified soy protein extraction by ultrasound. Chem Eng Process Process Intensif. 2017;113:94–101.

[52] Youssouf L, Lallemand L, Giraud P, et al Ultrasound-assisted extraction and structural characterization by NMR of alginates and carrageenans from seaweeds. Carbohydr Polym. 2017;166:55–63. 28385248 10.1016/j.carbpol.2017.01.041

[53] Luo J, Fang Z, Smith RL Ultrasound-enhanced conversion of biomass to biofuels. Prog Energy Combust Sci. 2014;41:56–93.

[54] Kumar AK, Sharma S Recent updates on different methods of pretreatment of lignocellulosic feedstocks: a review. Bioresources Bioprocess. 2017;4(1):7. 10.1186/s40643-017-0137-9 PMC5241333 28163994

[55] Roselló-Soto E, Galanakis CM, Brncic M, et al Clean recovery of antioxidant compounds from plant foods, by-products and algae assisted by ultrasounds processing. Modeling approaches to optimize processing conditions. Trends Food SciTechnol. 2015;42(2):134–149.

[56] Johner JCF, Hatami T, Meireles MAA Developing a supercritical fluid extraction method assisted by cold pressing for extraction of pequi (Caryocarbrasiliense). J Supercrit Fluids. 2018;137:34–39.

[57] Favareto R, Teixeira MB, Soares FAL, et al Study of the supercritical extraction of Pterodon fruits (Fabaceae). J Supercrit Fluids. 2017;128:159–165.

[58] Santos-Zea L, Gutiérrez-Uribe JA, Benedito J Effect of ultrasound intensification on the supercritical fluid extraction of phytochemical from Agave salmiana bagasse. J Supercrit Fluids. 2019;144:98–107.

[59] Conde-Hernández L, Espinosa-Victoria JR, Guerrero-Beltrán JA Supercritical extraction of essential oils of Piper auritum and Porophyllumriderale. J Supercrit Fluids. 2017;127:97–102.

[60] Benito-Román O, Rodríguez-Perrino M, Sanz MT, et al Supercritical carbon dioxide extraction of quinoa oil: study of the influence of process parameters on the extraction yield and oil quality. J Supercrit Fluids. 2018;139:62–71.

[61] Ferrentino G, Morozova K, Mosibo OK, et al Biorecovery of antioxidants from apple pomace by supercritical fluid extraction. J Clean Prod. 2018;186:253–261.

[62] Djas M, Henczka M Reactive extraction of carboxylic acids using organic solvents and supercritical fluids: a review. Sep Purif Technol. 2018;201:106–119.

[63] Guo M, Jin TZ, Yang R Antimicrobial polylactic acid packaging films against Listeria and Salmonella in culture medium and on ready-to-eat meat. Food Bioprocess Technol. 2014;7:3293–3307.

[64] Padil VVT, Senan C, Waclawek S, et al Bioplastic fibers from gum Arabic for greener food wrapping applications. ACS Sustainable Chem Eng. 2019;7:5900–5911.

[65] Varma RS Biomass-derived renewable carbonaceous materials for sustainable chemical and environmental applications. ACS Sustainable Chem Eng. 2019;(2019(7):6458–6470.

[66] Edlund U, Ryberg YZ, Albertsson AC Barrier films from renewable forestry waste. Biomacromolecules. 2010;(2010(11):2532–2538. 10.1021/bm100767g 20681735

[67] Wang H, Qian J, Fuyuan Ding F Emerging chitosan-based films for food packaging applications. J Agric Food Chem. 2018;66:395–413. 29257871 10.1021/acs.jafc.7b04528

[68] Lopez-Rubio A, Gavara R, Lagaron JM Bioactive packaging: turning foods into healthier foodsthrough biomaterials. Trends Food SciTechnol. 2006;17:567–575.

[69] Majid I, Nayik GA, Dar SM, et al Novel food packaging technologies: innovations and future prospective. J Saudi Soc Agri Sci. 2018;17:454–462.

[70] Guillard V, Gaucel S, Fornaciari C, et al The next generation of sustainable food packaging to preserve our environment in a circular economy context. Front Nutr. 2018;5:121. 30564581 10.3389/fnut.2018.00121 PMC6288173

[71] Ounkaew A, Kasemsiri P, Kamwilaisak K, et al Polyvinyl alcohol (PVA)/starch bioactive packaging film enriched with antioxidants from spent coffee ground and citric acid. J Polym Environ. 2018;26:3762–3772.

[72] Oliveira RA, De, Komesu A, Eduardo C, et al Challenges and opportunities in lactic acid bioprocess design—from economic to production aspects. Biochem Eng J. 2018 DOI:10.1016/j.bej.2018.03.003

[73] Petrides D Bioprocess design and economics In: Harrison RG, Todd PW, Petrides D, editors. Bioseparations science and engineering. US: Oxford University Press. 2013; p. 1–83. ISBN: 9780199731862

[74] Balasundaram B, Harrison S, Bracewell DG Advances in product release strategies and impact on bioprocess design. Trends Biotechnol. 2009;27(8):477–485. 19573944 10.1016/j.tibtech.2009.04.004

[75] Van Renterghem L, Roelants SLKW, Baccile N, et al From lab to market: an integrated bioprocess design approach for new-to-nature biosurfactants produced by Starmerella bombicola. Biotechnol Bioeng. 2018;115(5):1195–1206. 29288587 10.1002/bit.26539

[76] Schmideder A, Cremer JH, Weuster-Botz D Parallel steady state studies on a milliliter scale accelerate fed-batch bioprocess design for recombinant protein production with Escherichia coli. Biocatal Bioreactor Design. 2016;115(5):1195–1206. 10.1002/btpr.2360 27604066

[77] Silva MM, Rodrigues AF, Correia C, et al PLURIPOTENT STEM CELLS robust expansion of human pluripotent stem cells?: integration of bioprocess design with transcriptomic. Stem Cells Transl Med. 2015;4:731–742. 25979863 10.5966/sctm.2014-0270 PMC4479622

[78] Iqbal M, Tao Y, Xie S, et al Aqueous two-phase system (ATPS): an overview and advances in its applications. Biol Proced Online. 2016;1–18. DOI:10.1186/s12575-016-0048-8 27807400 PMC5084470

[79] Tyagi A, Kumar A, Aparna SV, et al Synthetic biology: applications in the food sector. Crit Rev Food Sci Nutr. 2016;56(11):1777–1789. 25365334 10.1080/10408398.2013.782534

[80] Heinemann M, Panke S Synthetic biology-putting engineering into biology. Bioinformatics. 2006;22(22):2790–2799. 16954140 10.1093/bioinformatics/btl469

[81] Tucker J, Zilinskas R The promise and the peril of synthetic biology. New Atlantis. 2006;12:25–45. 16832953

[82] Hobom B Surgery of genes. At the doorstep of synthetic biology. MedizineKlinik. 1980;75:14–21.

[83] Jagadeesana B, Gerner-Smidtb P, Allardc MW, et al The use of next generation sequencing for improving food safety: translation into practice. Food Microbiol. 2019;79:96–115. 30621881 10.1016/j.fm.2018.11.005 PMC6492263

[84] Zhaon H Synthetic biology tools and applications. Amsterdam, Boston: Book of Academic Press Elsevier; 2013 p. 3–327. ISBN: 978-0-12-394430-6.

[85] Guazzaroni ME, Silva-Rocha R, Ward RJ Synthetic biology approaches to improve biocatalyst identification in metagenomic library screening. Microb Biotechnol. 2015;8(1):52–64. 25123225 10.1111/1751-7915.12146 PMC4321373

[86] Heffernan C, Misturelli F The delivery of veterinary services to the poor: preliminary findings from Kenya report for DFID’s (Department for International Development) Animal Health Programme (AHP). Reading, UK: Livestock Development Group, The University of Reading; 2000.

[87] Nakaya N, Homma Y, Goto Y Cholesterol lowering effect of spirulina. J Dairy Sci. 1988;71:534–538.

[88] Jensen MK, Keasling JD Recent applications of synthetic biology tools for yeast metabolic engineering. FEMS Yeast Res. 2015;15:1–10. 10.1111/1567-1364.12185 25041737

[89] Anderson LA, Islam MM, Prather KLJ Synthetic biology strategies for improving microbial synthesis of “green” biopolymers. J Biol Chem. 2018;293(14):5053–5061. 29339554 10.1074/jbc.TM117.000368 PMC5892568

[90] Chappel J, Watters KE, Takahashi MK, et al A renaissance in RNA synthetic biology: new mechanisms, applications and tools for the future Author links open overlay panel. Curr Opin Chem Biol. 2015;28:47–56. 26093826 10.1016/j.cbpa.2015.05.018

[91] Li M, Borodina I Application of synthetic biology for production of chemicals in yeast Saccharomyces cerevisiae. FEMS Yeast Res. 2014;15:1–14. 25238571 10.1111/1567-1364.12213

[92] Gutiérrez D, Rodríguez-Rubio L, Martínez B, et al Bacteriophages as weapons against bacterial biofilms in the food industry. Front Microbiol. 2016;7(825):1–15. 27375566 10.3389/fmicb.2016.00825 PMC4897796

[93] Jagadeesan B, Gerner-Smidt P, Marc WA, et al The use of next generation sequencing for improving food safety: translation into practice. Food Microbiol. 2019;79:96–115. 30621881 10.1016/j.fm.2018.11.005 PMC6492263

[94] Jackson BR, Tarr C, Strain E, et al Implementation of nationwide real-time whole-genome sequencing to enhance Listeriosis outbreak detection and investigation. Clin Infect Dis. 2016;63:380–386. 27090985 10.1093/cid/ciw242 PMC4946012

[95] Gerner-Smidt P, Hyytia-Trees T, Barrett MD, et al Molecular source tracking and molecular subtyping in food microbiology: fundamentals and frontiers. 4th ed. Washington DC: ASM Press; 2013 p. 1059–1077.

[96] Timme RE, Rand H, Shumway MEK, et al Benchmark datasets for phylogenomic pipeline validation, applications for foodborne pathogen surveillance. PeerJ. 2017;5:e3893. 29372115 10.7717/peerj.3893 PMC5782805

[97] Besser J, Carleton HA, Gerner-Smidt P, et al Next-generation sequencing technologies and their application to the study and control of bacterial infections. Clin Microbiol Infect. 2018;24:335–341. 29074157 10.1016/j.cmi.2017.10.013 PMC5857210

[98] Weimer BC, Storey DB, Elkins CA, et al Defining the food microbiome for authentication, safety, and process management. IBM J Res Dev. 2016;60(5–6):1–13.

[99] IBM (2015). Consortium for sequencing the food supply chain: IBM research and mars tackle global health with food safety partnership, [online] Available: http://www.research.ibm.com/client-programs/foodsafety/.

[100] Belda I, Zarraonaindia I, Perisin M, et al Vineyard soil to wine fermentation: microbiome approximations to explain the “terroir” concept. Front Microbiol. 2017;8:821. 28533770 10.3389/fmicb.2017.00821 PMC5420814

[101] Meng F, Zhu X, Nie T, et al Enhanced expression of pullulanase in bacillus subtilis by new strong promoters mined from transcriptome data, both alone and in combination. Front Microbiol. 2018;9:2635. 30450090 10.3389/fmicb.2018.02635 PMC6224515

[102] Reddy CK, Pramila S, Haripriya S Pasting, textural and thermal properties of resistant starch prepared from potato (Solanum tuberosum) starch using pullulanase enzyme. J Food Sci Technol. 2015;52:1594–1601. 25745229 10.1007/s13197-013-1151-3 PMC4348285

[103] Elaziz MA, Hemdan AM, Hassanien A, et al Analysis of bioactive amino acids from fish hydrolysates with a new bioinformatic intelligent system approach. Sci Rep. 2017;7(1):10860. 28883610 10.1038/s41598-017-10890-1 PMC5589738

